# The Role of Neuropsychological Factors in Perceived Threat of SARS-CoV-2 in Healthy Ageing

**DOI:** 10.3390/ijerph18115847

**Published:** 2021-05-29

**Authors:** Massimo Bartoli, Nicola Canessa, Giuseppina Elena Cipriani, Stefano F. Cappa, Martina Amanzio

**Affiliations:** 1 Department of Psychology, University of Turin, 10124 Turin, Italy; massimo.bartoli@unito.it (M.B.); martina.amanzio@unito.it (M.A.); 2 ICoN Center, Scuola Universitaria Superiore IUSS, 27100 Pavia, Italy; nicola.canessa@iusspavia.it (N.C.); stefano.cappa@iusspavia.it (S.F.C.); 3 Istituti Clinici Scientifici Maugeri IRCCS, Cognitive Neuroscience Laboratory of Pavia Institute, 27100 Pavia, Italy; 4 Istituto di Ricovero e Cura a Carattere Scientifico (IRCCS) Mondino Foundation, 27100 Pavia, Italy; 5 European Innovation Partnership on Active and Healthy Ageing, 1049 Brussels, Belgium

**Keywords:** normal aging, perceived threat, cognitive functions, mood deflections, phenotypic frailty model

## Abstract

The COVID-19 pandemic is known to increase older adults’ vulnerability to adverse outcomes. Alongside increased physical frailty, anxiety symptoms associated with the risk of SARS-CoV-2 contagion appear to represent its most prominent ‘sequelae’. The attentional and linguistic resources required for decoding virus-related information may also influence the perceived threat of contagion. However, the possible role of neuropsychogeriatric factors on the latter dimension has never been assessed in a longitudinal study on the older population. To fill this gap, 50 healthy cognitively preserved older adults underwent a neuropsychological and physical frailty assessment before the pandemic (T0). Subsequently, they agreed to be interviewed and re-assessed during the lockdown (T1) and immediately after it (T2) through a longitudinal one-year study. Perceived threat of SARS-CoV-2 at T2 was predicted both by baseline anxiety and frailty scores, and by decreased performance in information processing speed and language comprehension tests. While confirming the joint role of frailty and anxiety, a moderation/interaction model showed that each of them was sufficient, at its highest level, to support the maximum degree of perceived threat of contagion. The contribution of neuropsychological factors to perceived threat of SARS-CoV-2 highlights their importance of tailoring information campaigns addressed to older people.

## 1. Introduction

The Severe Acute Respiratory Syndrome (SARS-CoV-2) is an emerging infectious disease that causes serious threats to the physical and mental health of the population.

The behavior of the general population, or of specific at-risk groups, such as aging subjects, can play an important role in both the spread and control of infectious diseases. The older population with a frailty status, which is characterized by a clinical history of polypathology, had higher mortality due to SARS-CoV-2 [1,2]. This increased susceptibility to infection is probably driven not only by comorbidity but also by reduced immunity due to the physiology of ageing [3].

Lockdown measures play a key role in containing SARS-CoV-2 infection. However, in the older population, aspects associated with isolation and social distancing are related to increased cognitive decline [4]. As a result of COVID-19 containment measures, older adults may experience physical and other mental health problems [5], such as depressive mood and anxiety-related fear of contagion [6]. In line with these aspects, a recent study [7] highlighted how both cognitive and physical issues related to the COVID-19 pandemic may play a key role in the novel “lockdown fatigue”. In particular, the authors found that pandemic fatigue was associated with decreased processing and walking speed and with mood changes in terms of depression.

While all these conditions represent the likely consequences of a pandemic, the variables driving modifications of behavior patterns—particularly concerning contagion prevention—also need to be considered and analyzed. One of the factors that can influence the willingness and motivation to adopt precautionary behavior is the perception of risk [8,9], i.e., the perceived personal vulnerability or likelihood of contracting a disease. Feeling vulnerable, together with realizing disease severity, can be jointly considered as “perceived threat”. People are expected to experience the highest perceived SARS-CoV-2 threat if they think that a likely infection will have serious health consequences. Indeed, people who are more vulnerable—such as older adults—present a higher level of fear [10] and anxiety [11] related to COVID-19.

On the other hand, some studies concerning outbreaks, such as the H5N1 and the current COVID-19 pandemic, found a lower risk perception in older adults than in younger people [12,13]. A recent study reported a lower perceived risk of being infected by COVID-19 in the oldest-old (75 years and older) than in the youngest-old (60–69 years) and middle-old (70–75 years) groups [14]. According to the authors, this result might reflect an age-related cognitive decline, particularly concerning executive control functioning, as risk-taking and risk perception are associated with monitoring abilities [15]. In light of this evidence, a reduction in perceived risk may lead older adults—who are more prone to unfavorable outcomes—to underestimate the importance of proper precautions to avoid COVID-19 infection.

To date, however, no longitudinal studies have analyzed the association among risk threat perception of SARS-CoV-2 contagion, physical-health status, cognitive functions, and mood deflections in cognitively preserved older adults during home confinement. These aspects should be further emphasized as personalized psychological interventions are found to play an important role in primary prevention and psychological well-being by reducing the negative impact on physical status, cognitive function, and mood disorders.

To fill this gap, we investigated the relationships among perceived risk of SARS-CoV-2 contagion, physical-cognitive functioning, and mood changes in cognitively preserved older adults, engaged as volunteers, and characterized by the presence of two or more age-related diseases (polypathology) and subsequent pharmacotherapy [16]. The availability of pre-pandemic data (at T0) of 50 subjects allowed us to investigate the extent to which perceived threat of SARS-CoV-2 was predicted by cognitive functioning, psychological state, and frailty determinants assessed with the phenotypic model [17], and by their interaction.

To the best of our knowledge, this is the first longitudinal study investigating: (a) the neuropsychological profile and frailty determinants of healthy older adults before the pandemic; (b) the perceived threat of SARS-CoV-2 contagion during the lockdown; and (c) whether the latter reflects the interacting effects of baseline variables concerning cognitive, psychological, and/or physical status.

Since perceived threat has been previously related to psychological issues, such as anxiety [18], and the latter may influence the occurrence of attentional and interpretation threat biases [19,20], also related to information about contagion, we expect that both psychological and cognitive factors might be useful in predicting perceived risk of contagion in our sample.

## 2. Materials and Methods

### 2.1. Participants

During the academic year 2018–2019, the creation of an active and healthy aging laboratory allowed us to carry out an in-depth neuropsychological assessment on members of the University of the Third Age (UNITRE) in Turin. Before the pandemic onset, these subjects attended specific teaching modules on cognition, physical exercise, nutrition, and social inclusion to achieve active and healthy aging. Among them, 81 agreed to participate in a longitudinal study with an expected duration of about one year. The subjects were aged 60 years or older in order to be classified as “older adults” [21], and fifty of them (40 females, age range 60–80) were selected due to the presence of polypathology and related pharmacotherapy [16]. However, the subjects were not taking psychotropic medications that could affect their cognitive functioning. Moreover, they did not complain of a subjective cognitive decline [22] and had not gone through any medical or neurological examination for suspected Mild Cognitive Impairment [23]. All participants were involved in social activities and characterized by functional autonomy. They performed an initial neuropsychogeriatric assessment (T0) aimed to detect any possible dysfunctions. We analyzed their cognitive functioning, physical health, and mood changes in terms of depression and anxiety.

During the pandemic, due to preventive measures, the subjects agreed to participate in the study remotely. They went through a semi-structured interview (T1) and a neuropsychological evaluation (T2) to identify any possible below cut-off scores.

The study was performed in accordance with the Declaration of Helsinki and approved by the Ethics Committee of the University of Turin before the pandemic (Prot. n. 10038) and after it (Prot. n. 151786). All participants gave written informed consent prior to the study.

### 2.2. Assessments

#### 2.2.1. Neuropsychogeriatric Assessment at the Baseline (T0)

From April to October 2019, all participants went through an extensive neuropsychiatric evaluation characterized by cognitive, functional, and behavioral tests. To prevent fatigue and lack of adherence to the tasks, three experienced neuropsychologists assessed the subjects in two different sessions, lasting about 90 min, held one day apart.

The Cumulative Illness Rating Scale (CIRS [24]) was used to collect information on clinical history, symptoms, signs, and chronic diseases.

We assessed global cognitive functioning adopting Addenbrooke’s Cognitive Examination—Revised version (ACE-R: [25]) and the Montreal Cognitive Assessment (MoCA [26]). Subsequently, we analyzed different cognitive domains: attention (attentional matrices [27]), language and fluencies (Token Test—TT [28]; Phonemic and Semantic Fluency [27]; Naming subtest of Aachener Aphasie Test Aphasia Test—AAT [29]), short-term memory (Digit Span and Corsi Test [27]), episodic long-term memory (Rey Memory Test and Short Story Recall [27]), problem-solving (Raven’s Colored Progressive Matrices—CPM-36 [27]), visuo-constructive abilities (Copy Design [30]), and executive functions (Trail Making Test—TMT [31]).

The Hamilton Rating Scale for Anxiety (HARS [32]) and the Beck Depression Inventory (BDI [33]) were used to assess anxiety and depression, respectively.

To estimate a physical frailty status, we adopted Fried’s phenotypic model [17]. This approach defines frailty as a pathophysiological syndrome characterized by five criteria: (a) unintentional weight loss; (b) poor grip strength; (c) self-reported exhaustion; (d) slow walking speed; and (e) low level of physical activity. If one or two determinants are present, we can identify a pre-frail person; if three or more, a frail one.

#### 2.2.2. Semi-Structured Interviews at T1

During the first Italian lockdown, from April to May 2020, all subjects previously recruited for the neuropsychological assessment at T0 were asked for their availability to participate in this study.

After agreeing, the participants went through a semi-structured interview to get information on their health conditions (i.e., symptoms related to SARS-CoV-2 infection and possible results of medical consultations, nasal swabs, or serological examinations), housing status, and the acceptance of the preventive measures to avoid contagion on the recommendations of the Italian Ministry of Health.

Before ending the interview, the subjects gave their availability for a new neuropsychological assessment.

#### 2.2.3. Neuropsychological Assessment at T2

This third phase was carried out from May to July 2020, during the Italian “phase 2” of quarantine, when restrictive measures introduced during the first lockdown were progressively relaxing. The neuropsychological assessment was as close as possible to T0 but characterized by limitations posed by a remote evaluation via video call.

We assessed cognitive functioning using ACE-R and Mini Mental State Examination (MMSE, [34]). To overcome visual issues, we presented visuo-spatial subsections in screen-sharing mode. In addition, we adopted the MoCA-Blind [35]. This blind version of the test was originally created for patients with visual disorders. As indicated on the official MoCA website [36], it is scored out of 22 and converted back to 30 as the original version.

BDI and HARS were used to assess possible mood changes. Finally, to analyze the socioeconomic status of the participants, we adopted the Four Factor Index of Social Status. Based on the educational level and the type of job of family members, this index (Hollingshead index, HI) was calculated considering the last employment of these retired people. The HI score ranges from 8 to 66 points and identifies 5 different social strata [37,38].

##### Risk of Contagion Assessment

To detect participants’ perceived risk of contracting SARS-CoV-2, we used a modified version of the Risk Perception of Infectious Diseases Questionnaire [9,39,40]. This tool was based on a previously developed SARS risk perception questionnaire [39] and focused on risk perception and perceived severity of SARS, and other (infectious) diseases (i.e., flu [40]).

The questionnaire is divided into 5 sections, requiring participants to report the perceived: (1) severity (i.e., how serious it would be for them to get the disease); (2) vulnerability or personal risk (i.e., how likely they were to contract the disease); (3) comparative risk (i.e., whether they were more or less likely to contract the disease than individuals of their own sex and age); (4) response efficacy (i.e., to what extent they thought people could take effective actions to prevent the onset or contagion of the disease); (5) self-efficacy (i.e., to what extent they considered themselves capable of taking effective actions to prevent the disease onset or contagion).

In the original version, some sections (e.g., personal and comparative risk) were assessed on a 5-point Likert scale, while others (e.g., severity) were evaluated on a 10-point scale [8]. In our study, participants were asked to answer on a 10-point Likert-type scale in all sections to uniform the attribution of scores.

In line with a previous study [9], we obtained the “perceived threat” value by multiplying the measures of perceived severity and vulnerability. In the original version, these two categories were measured respectively on 1–10 and 1–5 point scales, with the former being halved to make their values comparable. In the present version, this operation was not performed because the scores of all 5 questionnaire sections were assessed on a 1–10 scale. To normalize the distribution of this new variable, a square root transformation was performed giving a measure of perceived threat on a scale of 1 (low) to 10 (high).

In the present study, we have considered only the “perceived threat” section of the Risk Perception of Infectious Diseases Questionnaire. Particularly, we focused our attention on the data concerning the COVID-19 pandemic and a possible emerging new virus. In fact, our aim was to understand the extent to which older adults perceived COVID-19 as threatening in relation to their physical, cognitive, and mental health status. In addition, given the sudden onset of the current pandemic, we wanted to verify whether these aspects, assessed at T0, could affect the perceived threat of the risk of a possible emerging virus.

### 2.3. Data Analyses

#### Multiple Regression Analyses

At first, we have taken into account baseline physical, cognitive, and mental health status variables (collected at T0), trying to identify those associated with the perceived threat of SARS-CoV-2 infection (assessed at T2). Specifically, we hypothesized that a higher perceived threat of contagion might be related to an increased physical pre-frailty status and anxiety symptoms. Moreover, from a cognitive perspective, attentional and linguistic resources required for decoding virus-related information may also influence the perceived threat of contagion.

To this end, we first assessed variables that showed a significant linear relationship with the ‘Perceived Threat’. The results of the correlation analyses confirmed our initial hypothesis: perceived threat of contagion was associated with higher anxiety (HARS) and higher pre-frailty status. In addition, greater susceptibility to threat perception was associated with lower performance in information processing speed (TMT-A score) and language resources (TT score).

On this basis, we performed two multiple regressions to estimate the extent to which the perceived risk of contracting SARS-CoV-2, or a new virus, was explained by socio-demographic variables (age, gender, education), anxiety (HARS) and frailty status, attentional (TMT-A score) and linguistic comprehension (TT) performance.

A preliminary check of assumptions confirmed the lack of multicollinearity among explanatory variables (maximum variance inflation factors (VIFs) = 2.54 and 2.08, respectively), as well as the normal distribution (Kolmogorov–Smirnov tests, *p* > 0.2 in both cases) and independence (Durbin-Watson = 1.851 and 1.891, respectively) of residuals.

Based on the results of multiple regressions, we assessed a moderation model to test interaction between anxiety and frailty on the perceived risk of contracting SARS-CoV-2 (while modeling age, gender, and educational level as nuisance variables). We used the PROCESS macro (v.3.5) for SPSS (v.23, IBM, Armonk, NY, USA) to test Hayes’ [41] model 1 (moderation), through conditional process analysis based on Ordinary Least Squares (OLS) regression, using bootstrapping resampling (50,000 samples) to generate confidence intervals for direct and moderated effects. Interaction variables were centered (to a mean of 0) before entering the analyses and Johnson and Neyman’s [42] approach was used to compute the range of significance and simple slopes for the interaction analyses, which were assessed 1 standard deviation below and above the mean. The statistical threshold was set at *p* < 0.05 (two-tailed).

## 3. Results

### 3.1. T0 Main Results

The mean scores obtained at T0 on cognitive tests and behavioral scales are shown in Table 1. Subjects had a MMSE raw score ≥ 27. Participants performed below the reference cut-off value on some tests: Digit span (4%); Attentional matrices (2%); Phonetic fluency test (2%); Corsi test (2%); Rey memory test instant recall (2%), and delayed recall (2%). However, the percentage of under cut-off scores is consistent with the margin of error in tests administered to the normative population. Moreover, they displayed mood changes concerning depression (BDI = 28%) and anxiety (HARS = 4%).

According to Fried criteria, 68% and 32% of participants could be classified as robust and pre-frail, respectively. Particularly, the latter presented a decrease in handgrip strength and walking speed (26% and 2%, respectively).

### 3.2. T1 Main Results

The socio-demographic characteristics of the subjects and the percentages on the use of protective devices are reported in Table 2.

According to the outcome of semi-structured interviews, 62% of the subjects lived with at least one person (in most cases the spouse), while the remaining 38% lived alone.

Subjects’ high awareness of the risk of contagion was confirmed by their compliance with most of the prevention behaviors indicated by the Italian Ministry of Health [43] (see Table 3). In particular, 96% of the participants stated they had used masks, had respected the minimum safety distances, and had often sanitized their hands (with soap and water or disinfectant solutions). Moreover, 90% used latex gloves outside, while 84% avoided crowded places. Furthermore, none of the participants declared to have developed symptoms associated with COVID-19 and none of them was diagnosed with COVID-19 infection or was hospitalized.

### 3.3. T2 Main Results

Table 4 reports the socio-economic status (SES) of our sample and the mean scores obtained by the subjects on the cognitive screening test. Specifically, they performed well on all cognitive tests (MMSE, ACE-R, and MoCA Blind). As to mood assessment, there were below cut-off scores on BDI (28%), and HARS (10%). It should be noted that the percentage of subjects with anxiety-related issues had increased from T0 to T2 (from 4% to 10% of the sample) but no differences were found in the percentage of subjects with mood deflection compatible with depression.

According to the Hollingshead Index (HI), most of the sample belonged to the medium-high SES (90%). Particularly, 8% of the subjects fell into the highest social stratum, 48% in the second, 34% in the third, 10% in the fourth, and none of them in the lowest one.

Analyzing COVID-19 perceived threat as a dependent variable, fifty percent of variability in the perceived risk of contracting SARS-CoV-2 was associated with a combination of measures related to cognitive, psychological, and physical status. Namely, higher risk perception was predicted by: (a) decreased information processing speed (TMT-A score) and linguistic comprehension (TT) performance; (b) increased anxiety on HARS, and frailty status in terms of the phenotypic model (*p* = 0.000002; see Table 5).

Furthermore, analyzing the risk of infection by a ‘new virus’ as a dependent variable, 40% of the variance in the perceived risk of contracting other (emerging) infectious diseases was also explained by decreased information processing speed (TMT-A score) and linguistic comprehension (TT) performance, and by increased anxiety on HARS (*p* = 0.00003; see Table 6). This dependent variable was not significantly predicted by frailty. Therefore, it seems to be more strongly associated with perceived risk of COVID-19 contagion.

Based on the latter finding, and on the role of anxiety in risk perception [18,20,44], we selected these variables as predictors of a moderation model assessing the effect of baseline frailty and anxiety levels, and their interaction, on risk perception of SARS-CoV-2 at T2 (see Table 7). Indeed, we found that the perceived threat of contracting SARS-CoV-2 was predicted by higher frailty (*p* = 0.0034) and anxiety (*p* = 0.0075), while additionally showing an interaction between these two predictors (*p* = 0.0338).

**Figure 1:**
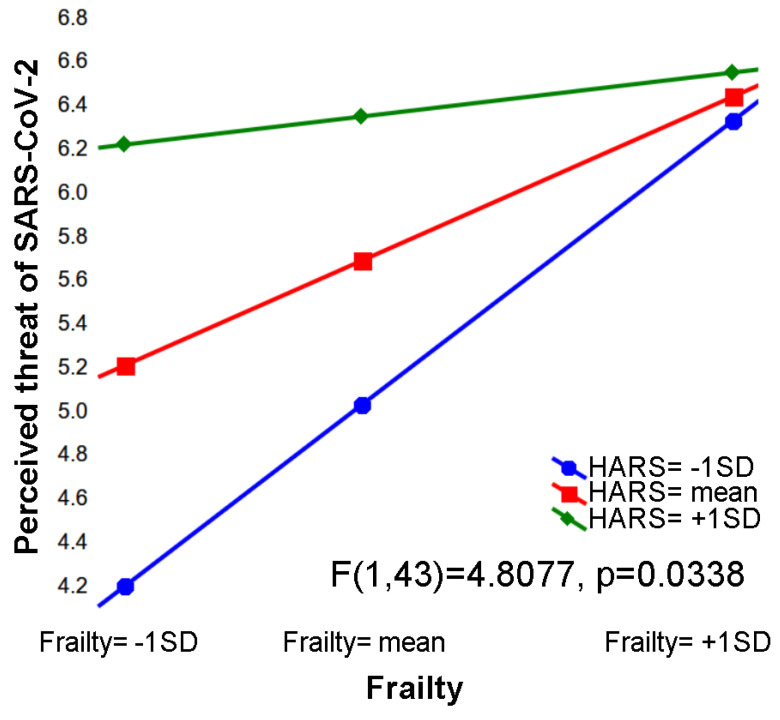
The figure depicts the interacting effects of physical frailty (based on Fried’s phenotypic model) and anxiety (HARS) at T0 on perceived threat of SARS-CoV-2.

As shown in Figure 1, the perceived threat of SARS-CoV-2 contagion is maximal at the highest anxiety level regardless of frailty, and at the highest frailty level regardless of anxiety. Therefore, each of these predictors is sufficient, at its highest level, to support the maximum degree of perceived threat (Figure 1). Instead, when their values are average or under-the-mean, both predictors are required to promote perceived threat, which indeed is minimum at the lowest frailty and anxiety levels.

## 4. Discussion

To the best of our knowledge, this is the first published study that has monitored subjects in healthy cognitive aging for more than one year, before and after extremely restrictive measures due to the COVID-19 pandemic (i.e., from April 2019 to July 2020). This longitudinal design has provided a unique opportunity to investigate whether, and to what extent, the perceived threat of contracting SARS-CoV-2 is explained by pre-existing individual differences concerning physical pre-frailty, cognitive performance and mood deflections. Indeed, unlike related studies on the effect of the pandemic, T0 data allowed us to unveil novel associations between pre-COVID-19 neuropsychological variables and perceived risk of contagion in the last stage of the lockdown.

Although most of the participants were robust at T0, some of them were pre-frail. Only a few minor neuropsychological deficits were observed in cognitive functioning and in line with the margin of error in tests administered to the normative population. Moreover, some participants showed mood changes in terms of depression and anxiety but these deflections were not significant from a mental health perspective.

Concerning the T1 results, almost all subjects complied with the precautions recommended by the Italian Ministry of Health [43] to prevent infection with SARS-CoV-2 and showed correct behaviors regarding the prevention of infection. It is noteworthy that almost the whole sample (90%) belonged to an upper-middle social class, according to HI. Combined with the high level of education, such aspects make clear the peculiarity of this population, characterized by social resources, which have enabled them to take the necessary precautions to avoid the risk of contagion. In fact, risk perception can be influenced by several aspects, including knowledge of the virus [44] and the socio-economic status. Previous studies [45,46] have shown that older, socio-economically disadvantaged, and poorly-educated individuals are less informed about COVID-19 spreading and, therefore, do not take adequate precautions.

The T2 data collected immediately after the isolation period showed neither a significant worsening of cognitive functions nor mood deflections in terms of depression. However, the level of anxiety increased during the COVID-19 pandemic, from 4% to 10%.

Multiple linear regression analyses showed that perceived risk of SARS-CoV-2 at T2 was predicted by worse performance on the information processing speed component and on language comprehension, as well as higher levels of pre-frailty and anxiety, at T0. These cognitive difficulties may hamper the correct interpretation of the information provided on the risk of infection, leading to attentional threat bias, further increasing anxiety [19]. Moreover, conflicting and confusing sources of information about the ongoing pandemic, in terms of ‘infodemia’, could exacerbate psychological distress in terms of anxiety and fear of contagion [47,48]. This would be even more pronounced in language processing in normal aging. Older subjects’ comprehension, even in the normative group, may also be less accurate due to an age-related slowdown in processing time, which increases in syntactically complex sentences. This pattern suggests an age-related decrease in parsing and interpretation efficiency suggesting how task-related operations are related to overall processing speed and working memory [49].

In addition, the presence of a frailty state may reasonably lead to a greater perceived threat, as mortality rates from Covid-19 are higher in older adults suffering from this condition [50].

In line with these considerations, the perceived threat caused by a new emerging virus, assessed at T2, was explained by reduced baseline performance in tasks of information processing speed and in linguistic comprehension (TMT-A and TT, respectively), and an increased level of anxiety (HARS), but not by frailty status. An unknown risk is usually perceived as more frightening [44]; thus, a high level of anxiety at baseline may intensify the sense of danger. Furthermore, anxiety may influence attentional abilities [20] but also the comprehension and the interpretation of stimuli [19].

Since multiple regression models showed that frailty was related only to the perceived threat of SARS-CoV-2, we hypothesized that this factor, along with the anxiety status assessed at baseline, might play an important role in explaining perceived risk. Indeed, a moderation model showed an interaction between these two independent variables in predicting SARS-CoV-2 perceived threat. Particularly, at their highest level, both frailty and anxiety are sufficient to support the maximum degree of risk perception, on the other hand, when their values decrease, both are required to promote SARS-CoV-2 threat perception.

### Limitation Section

Although the present study was carefully designed and achieved its purposes, some limitations should be addressed.

Firstly, an important aspect to be clarified concerns the scale used to assess threat perception. Even though the evaluation of “Perceived Threat” was performed indirectly, multiplying the measures of perceived “severity” and “vulnerability” of the Risk Perception of Infectious Diseases Questionnaire, it should be noted that this methodology has been validated and used in previous studies, i.e., [9]. In particular, the “Perceived Threat” scale was considered a reliable tool for assessing risk perception of infection due to a very contagious and dangerous virus.

Secondly, our subjects were enrolled at the University of the Third Age, which promotes learning during aging. For this reason, they represent a very distinctive sample of older adults, characterized by a medium-high socioeconomic status and a medium-high level of education.

Finally, a sample size of 50 participants represents a limitation to this study; however, this population was studied in-depth through a neuropsychogeriatric assessment before the COVID-19 pandemic (T0) and during the first lockdown, both with strong restrictive measures (T1) and when they were eased (T2).

## 5. Conclusions

To the best of our knowledge, this is the first study examining the association between perceived threat of SARS-CoV-2 during the lockdown and a set of cognitive, psychological, and physical variables measured before and during restrictive lockdown measures, in cognitively normal aging subjects. Therefore, it is important to underline how UNITRE healthy participants represent a very distinctive reference sample to implement possible primary prevention pathways on older adults, with a particular focus on early determinants that may affect well-being in the current pandemic.

Our results showed, on the one hand, how physical frailty and mood deflection in terms of anxiety and, on the other hand, how cognition involved in information processing speed tasks and language comprehension, could influence the perceived threat of SARS-CoV-2 infection risk. The evidence that neuropsychogeriatric factors contribute to the perception of risk of an infectious disease shows the need to consider these variables.

Particular attention should be paid to frailty, as the pandemic lifestyle, characterized by reduced social interaction and a decrease in physical activities, may lead to the so-called “Corona-Frailty” [51]. Moreover, the level of anxiety should be considered as it seems to modulate the perception of fear concerning COVID-19 infection.

The evidence that neuropsychological factors contribute to the perceived threat of an infectious disease (i.e., new virus) shows the need to consider these variables when planning information campaigns in older people, with the aim of achieving favorable changes in public behavior.

The present study emphasizes the contribution of neuropsychological factors to perceived threat of SARS-CoV-2 highlighting the importance of tailoring information campaigns addressed to older people.

The presence of pre-pandemic neurocognitive measures has provided a unique opportunity to investigate which facets of psychological, physical, and cognitive status predict a crucial variable such as perceived threat of SARS-CoV-2 in the aftermath of the lockdown. Furthermore, the COVID-19 containment measures, constrained to perform T2 neuropsychogeriatric assessment via selected neuropsychological, tests as suitable for remote administration.

## Figures and Tables

**Table 1:** Demographic and neuropsychological characteristics (T0).

	N	M	SD	Cut-Off
**Demographic characteristics**				
Subjects	50			
Gender [Male/Female]	10/40			
Age [years]		68.32	5.75	
Education [years]		12.84	2.79	
**Neuropsychological assessment**				
Mini Mental State Examination		29.12	0.98	≥23.8
Addenbrooke’s Cognitive Examination—Revised version		92.80	4.27	≥79 (<75 years old);
				≥60 (>75 years old)
Montreal Cognitive Assessment		25.18	2.29	≥17.363
Rey Memory Test—15 instant words		44.20	8.39	≥28.53
Rey Memory Test—15 delayed words		9.36	2.44	≥4.69
Babcock Short Story recall test		18.05	4.15	≥8
Digit span forward		6.20	1.05	≥4.25
Corsi Test		5.32	0.96	≥3.75
Phonemic verbal fluency		41.44	10.99	≥17.35
Semantic verbal fluency		26.15	5.21	≥7.25
Token Test		33.34	2.07	≤29
Aachener Aphasie Test		116.02	2.55	≥106
Coloured Progressive Matrices-36		32.26	3.11	≥18.96
Attentional Matrices		49.62	6.96	≥30
Coping design—without programming elements		10.32	1.22	≥7.18
Coping design—with programming elements		68.42	1.59	≥61.85
Trail Making Test-part A		39.58	12.01	≤94
Trail Making Test-part B		93.04	29.24	≤283
Trail Making Test B-A		53.47	25.75	≤187
**Neuropsychiatric assessment**				
Beck Depression Inventory		7.62	5.82	≤9
Hamilton Rating Scale for anxiety		5.34	3.94	≤14
**Functional assessment**				
CIRS—severity index		1.25	0.15	
CIRS—comorbidity index		0.80	0.66	

Note: N = number. M = mean. SD = standard deviation. CIRS = Cumulative Illness Rating Scale.

**Table 2:** Socio-demographic characteristics, pandemic-related information, and changes in habits of the study population at T1.

	N	%	M	SD
**Socio-demographic characteristics**				
Subjects	50			
Gender [M/F]	10/40			
Age [years]			70.02	5.69
Education [years]			12.84	2.76
Housing status [single/cohabitant]	19/31			
**Information pandemic-related**				
Health conditions				
symptoms COVID-19 related		18%		
medical examination		8%		
nasal swap		2%		
positivity to COVID-19		0%		
Main preventive measures				
using face masks		96%		
wearing latex gloves		90%		
keeping safety distance		96%		
washing hands		96%		
avoiding crowded places		84%		

Note: N = number. M = mean. SD = standard deviation. M = male. F = female.

**Table 3:** Recommendations of the Italian Ministry of Health to contain the spread of coronavirus.

Wash your hands frequently with soap and clean surfaces with chlorine or alcohol-based disinfectants.
Avoid crowded places and keep a distance of at least one meter from others.
Avoid touching your eyes, nose, and mouth.
Stay at home if you are elderly or have a weakened immune system.
Avoid handshakes, hugs, and sharing bottles and glasses with others.
Cover mouth and nose with a disposable tissue when you sneeze or cough. If you do not have a tissue, use your bent elbow.
If you have flu-like symptoms, stay at home, do not go to the emergency room or doctor’s office, but call your general practitioner, pediatrician, primary care out-of-hours service (“guardia medica”) or regional information hotline.

**Table 4:** Socio-demographic characteristics and neuropsychological assessment (T2).

	N	M	SD	Cut-Off
**Socio-demographic characteristics**				
Subjects	50			
Gender [Male/Female]	10/40			
Age [years]		70.04	5.70	
Education [years]		12.84	2.76	
SES (Hollingshead Index)		41.62	9.14	
social stratum 66–55		8%		
social stratum 54–40		48%		
social stratum 39–30		34%		
social stratum 29–20		10%		
social stratum 19–18		0%		
**Neuropsychological assessment**				
Mini-Mental State Examination		29.44	0.67	≥23.8
Addenbrooke’s Cognitive Examination—Revised version		95.04	3.37	≥79 (<75 years old);
				≥60 (>75 years old)
Montreal Cognitive Assessment		27.02	2.61	≥17.363
**Neuropsychiatric assessment**				
Beck Depression Inventory		7.70	6.10	≤9
Hamilton Rating Scale for anxiety		6.64	5.45	≤14
**Perceived Threat assessment**				
COVID-19		5.65	1.88	
New Virus		5.01	2.55	

Note: N = number. M = mean. SD = standard deviation. SES = Socioeconomic status.

**Table 5:** Multiple linear regression analysis for predicting the perceived threat of SARS-CoV-2 contagion.

Predictors	B	SE	*t*	*p*	R-Square	F (4,45)	*p*
TMT-A	−0.344	0.106	−3.231	0.002	0.506	11.536	<0.0001
Token Test	−0.330	0.105	−3.145	0.002			
HARS	0.405	0.105	3.839	<0.001			
Frailty	0.269	0.106	2.528	0.015			

Note: B = Regression coefficient. SE = Standard Error. TMT-A = Trail Making Test—part A. HARS = Hamilton Rating Scale for anxiety. The table reports the significant predictors of the perceived threat of SARS-COV-2 based on linear multiple regressions. The statistical values of both the whole model and the single predictors are reported.

**Table 6:** Multiple linear regression analysis for predicting the perceived threat of a new, emerging virus contagion.

Predictors	B	SE	*t*	*p*	R-Square	F (3,46)	*p*
TMT-A	−0.306	0.115	−2.672	0.010	0.400	10.251	<0.0001
Token Test	−0.355	0.114	−3.114	0.003			
HARS	0.441	0.114	3.849	<0.001			

Note: B = Regression coefficient. SE = Standard Error. TMT-A = Trail Making Test—part A. HARS = Hamilton Rating Scale for anxiety. The table reports the significant predictors of the perceived threat of a new, emerging virus based on linear multiple regressions. The statistical values of both the whole model and the single predictors are reported.

**Table 7:** Moderation analysis for predicting the perceived threat of SARS-CoV-2 at T2 based on frailty and anxiety, and their interaction, at T0.

Model Summary						
R	R^2^	MSE	F	df1	df2	*p*
0.6009	0.3611	2.5642	4.0512	6	43	0.0026
						
**Model**						
	coeff	se	t	*p*	LLCI	ULCI
constant	8.583	3.6846	2.3294	0.0246	1.1522	16.0138
Frailty	1.3324	0.4302	3.0971	0.0034	0.4648	2.2
Anxiety	0.1677	0.0597	2.8085	0.0075	0.0473	0.2881
Frailty x Anxiety	−0.2476	0.1129	−2.1927	0.0338	−0.4754	−0.0199
gender	−0.3951	0.6112	−0.6464	0.5214	−1.6276	0.8375
age	−0.0098	0.0421	−0.2334	0.8165	−0.0948	0.0751
education	−0.1175	0.0889	−1.3216	0.1933	−0.2969	0.0618
						
**Test(s) of highest order unconditional interaction(s):**						
	R2-chng	F	df1	df2	*p*	
Frailty x Anxiety	0.0714	4.8077	1	43	0.0338	
						
**Conditional effects of frailty at values of anxiety**						
HARS_T0	Effect	se	t	*p*	LLCI	ULCI
−3.9363	2.3071	0.6833	3.3767	0.0016	0.9292	3.6851
0	1.3324	0.4302	3.0971	0.0034	0.4648	2.2
3.9363	0.3576	0.5464	0.6545	0.5163	−0.7443	1.4596

Note: MSE = Mean Squared Error; df = degrees of freedom; LLCI = lower level of confidence interval; ULCI = upper level of confidence interval; coeff = coefficient; HARS_T0 = Hamilton Rating Scale for anxiety, assessed at T0; se = standard error. The table reports the results of a moderation model testing the effect of frailty and anxiety at T0, and their interaction, on the perceived threat of SARS-COV-2 at T2. The statistical values of both the whole model and the single predictors, alongside their interaction, are reported.

## Data Availability

Data is provided on request due to restrictions (e.g., privacy or ethical).
